# Molecular mechanism and application of emerging technologies in study of bacterial persisters

**DOI:** 10.1186/s12866-024-03628-3

**Published:** 2024-11-16

**Authors:** Shuo Yuan, Yamin Shen, Yingying Quan, Shuji Gao, Jing Zuo, Wenjie Jin, Rishun Li, Li Yi, Yuxin Wang, Yang Wang

**Affiliations:** 1 College of Animal Science and Technology, Henan University of Science and Technology, Luoyang, 471000 China; 2 Henan Provincial Engineering Research Center for Detection and Prevention and Control of Emerging Infectious Diseases in Livestock and Poultry, Luoyang, 471003 China; 3 College of Life Science, Luoyang Normal University, Luoyang, 471934 China

**Keywords:** Antibiotic persistence, Persisters, Tolerance, Resistance, Persistent infections, Quorum sensing systems, Biofilms

## Abstract

Since the discovery of antibiotics, they have served as a potent weapon against bacterial infections; however, natural evolution has allowed bacteria to adapt and develop coping mechanisms, ultimately leading to the concerning escalation of multidrug resistance. Bacterial persisters are a subpopulation that can survive briefly under high concentrations of antibiotic treatment and resume growth after lethal stress. Importantly, bacterial persisters are thought to be a significant cause of ineffective antibiotic therapy and recurrent infections in clinical practice and are thought to contribute to the development of antibiotic resistance. Therefore, it is essential to elucidate the molecular mechanisms of persister formation and to develop precise medical strategies to combat persistent infections. However, there are many difficulties in studying persisters due to their small proportion in the microbiota and their non-heritable nature. In this review, we discuss the similarities and differences of antibiotic resistance, tolerance, persistence, and viable but non-culturable cells, summarize the molecular mechanisms that affect the formation of persisters, and outline the emerging technologies in the study of persisters.

## Introduction

In bacterial populations, a small proportion of bacteria will enter a metabolically inactive or dormant state, either spontaneously or induced by environmental stress, and show high tolerance to a wide range of drugs [1]. In 1944, Bigger discovered that penicillin could not completely kill staphylococci in experiments and that the surviving colonies could still be effectively killed by penicillin after recultivation, leading to the first discovery of this subgroup, which he named “persisters” to distinguish them from drug-resistant mutants [2]. However, at that time, antibiotics were effective in treating bacterial infections, and new antibiotics were constantly being developed; the discovery of persisters did not receive attention. The presence of persisters has been found in a variety of bacteria (Table 1). The emergence of chronic infectious and inflammatory diseases has been on the rise since the beginning of the 21st century and has become a major public health problem worldwide [3]. There is increasing evidence of a strong association between persistent and persistent infections. For example, in a patient with cystic fibrosis, late isolates of *Pseudomonas aeruginosa* (*P. aeruginosa*) were 100-fold more persistent than early isolates [4]. Similarly, in *Escherichia coli* (*E. coli*), *hipA* mutant strains with high persistence were selected in patients with recurrent urinary tract infections [5]. In animal models, Patrick Kaiser and colleagues [6]used ciprofloxacin in *Salmonella*-infected mice. The results showed that high doses of ciprofloxacin killed most *Salmonella* within a few hours. However, at the end of the subsequent 10 days of treatment, approximately 10–20% of the bacteria remained viable in the cecum draining lymph node. In vitro experiments showed that the surviving cells were genetically susceptible to ciprofloxacin but significantly more tolerant [6]. Similar results were found in animal models of other studies [7]. These converging results suggest that persisters can cause chronic infections and are also selected for in the treatment of infections. In particular, recent results suggest that persistence emerges earlier than resistance under antibiotic treatment and may promote the development of resistance [8, 9].

While persisters pose a significant public health and safety threat, they have also attracted much scientific interest in their characteristics and formation mechanisms. However, the percentage of persisters in the population is tiny, the offspring cannot inherit the persistent phenotype, and it is not easy to maintain the persistent phenotype during the research process, so there is no practical method to enrich the isolation of persisters, which is a significant obstacle to the research. In this paper, we present the phenotypic characteristics of persisters and the similarities and differences with a variety of other drug-resistant bacterial forms, which will help readers to fully understand persisters. Second, this paper reviews the recently reported mechanisms that induce persister formation and proposes a model in which the biofilm promotes persister formation and protects them, ultimately leading to persistent infections. Finally, we discuss biological research methods applied to the study of persisters at different scales according to the characteristics of persisters, and to the best of our knowledge, this is the first article to do so.

**Table 1:** Bacteria reported to form persisters and their related diseases

Bacteria	Disease	Tools to analyze persisters^a^	References
*Mycobacterium abscessus*	Pneumonia	CFU	[10]
*Mycobacterium smegmatis*		microfluidic culture, time-lapse microscopy, and CFU	[11]
*Mycobacterium* *tuberculosis*	Tuberculosis	CFU, SCRS, and transcriptomics	[12]
*Mycobacterium marinum*	Wound festers	CFU and microscopy	[13]
*Mycobacterium bovis*	Bovine tuberculosis	CFU	[14]
*Acinetobacter baumannii*	Respiratory and urinary tract infections	CFU	[15]
*Bacillus subtilis*		CFU and IFC	[16–18]
*Borrelia burgdorferi*	Lyme disease	CFU and microscopy	[19, 20]
*Brucella sp*	Brucellosis	CFU	[21, 22]
*Burkholderia cepacia*	Septicemia, pneumonia	CFU	[23]
*Burkholderia pseudomallei*	Septicemia	CFU	[23]
*Vibrio cholerae*	Cholera	CFU and transcriptomics	[24]
*Edwardsiella tarda*	Gastroenteritis	CFU	[25]
*Escherichia coli*	Diarrhea, extraintestinal infection, extraintestinal infection	STM, CFU, SCRS, FACS, IFC, microfluidic culture, and microscopy	[26, 27, 18, 28]
*Enterobacter aerogenes*	Urinary tract infection, respiratory tract infection	CFU	[29]
*Enterococcus faecium*	Neonatal sepsis, urinary tract infection	CFU	[29]
*Klebsiella pneumoniae*	Pneumonia, bacteremia	CFU	[30]
*Streptococcus mutans*	Tooth decay	CFU and STM	[31]
*Streptococcus suis*	Meningitis, bacteremia	CFU	[32]
*Streptococcus* *pyogenes*	Pharyngotonsillitis, pneumonia	CFU	[33]
*Streptococcus pneumoniae*	Pneumonia, otitis media	CFU	[34, 35]
*Candida albicans*	Oral, gastrointestinal, and vaginal infections, Septicemia	CFU	[36, 37]
*Candida glabrata*		CFU	[38, 39]
*Candida tropicalis*		CFU and microscopy	[39, 40]
*Staphylococcus aureus*	Osteomyelitis, endocarditis, infections of indwelling devices, and wound infections	CFU, FACS, and STM	[2, 41, 42]
*Staphylococcus saprophyticus*	Urinary tract infection	CFU and STM	[43]
*Staphylococcus epidermidis*	Pyogenic infection, septicemia	CFU	[44]
*Listeria monocytogenes*	Listeriosis	CFU and microscopy	[45, 46]
*Salmonella Typhimurium*	Acute gastroenteritis	CFU	[47]
*Pseudomonas aeruginosa*	Chronic suppurative otitis media	CFU	[48]

^a^Abbreviations: CFU, colony-forming unit; SCRS, Single-cell Raman spectroscopy; FACS, fluorescence-activated cell sorting; IFC, imaging flow cytometry; STM, signature-tagged mutagenesis.

## How bacteria resist antibiotics

Since their introduction, antibiotics have been an effective weapon against various bacterial infections. However, with the massive use of antibiotics, bacteria have gradually found many different ways to respond to ensure the continuation of populations, and the development of new antibiotics has many difficulties, so elucidating the multiple forms of bacterial resistance to antibiotics and making targeted responses to develop new precision medical strategies are essential measures to address global public health problems.

### Persister cell

**Fig. 1:**
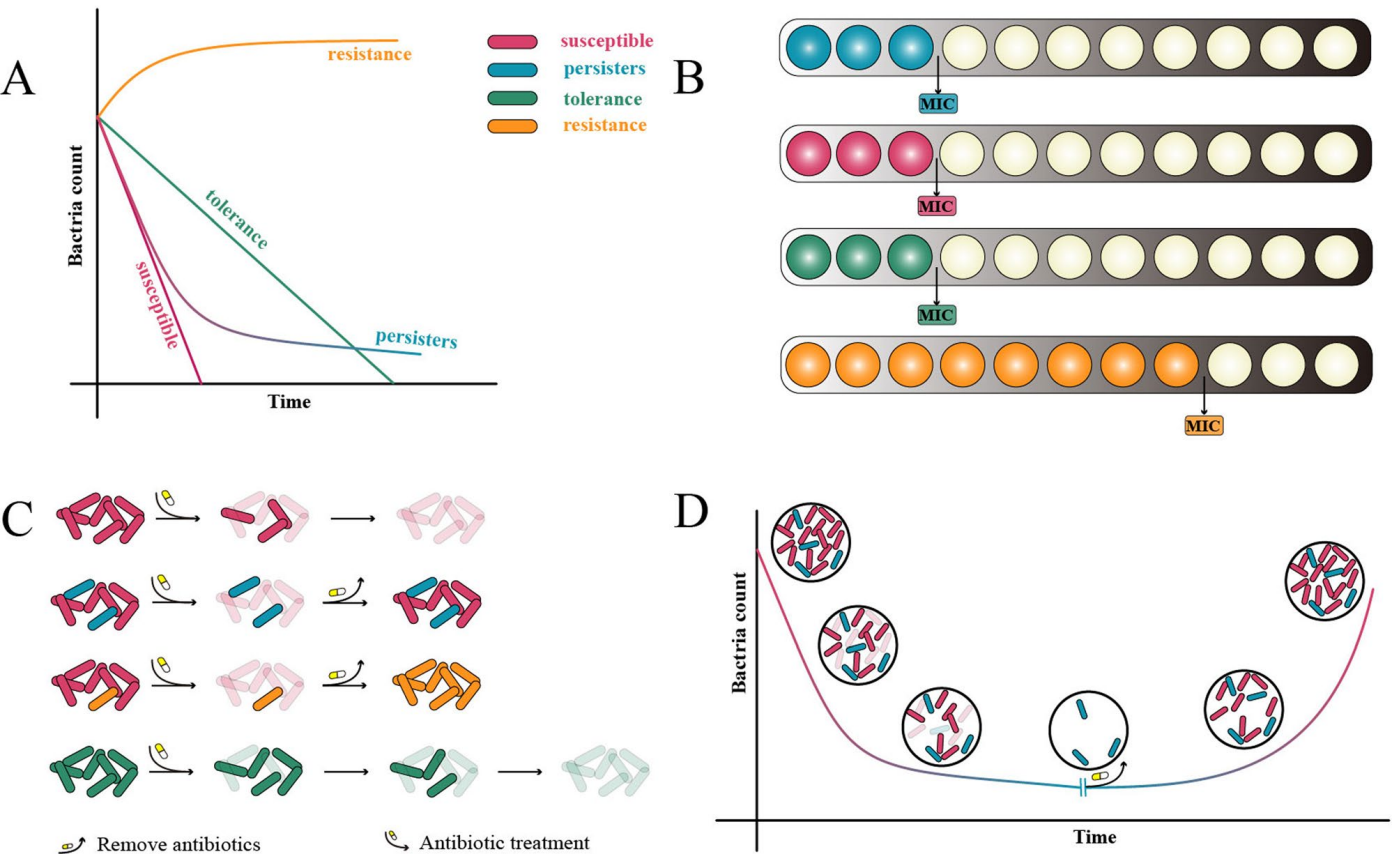
Physiological characteristics of resistant, tolerant, and persister cells. (**A**) Curves of the number of viable susceptible, persistent, tolerant, and resistant cells over time in the presence of lethal concentrations of antibiotics. (**B**) Persistent and tolerant bacteria had the same MIC as susceptible bacteria, while the MIC of resistant bacteria was significantly higher. The transition from a white to a black background represents a gradual increase in antibiotic concentration. (**C**) The process by which susceptible, tolerant, persistent, and resistant bacteria die when antibiotics are added and recover after removing antibiotics. Translucent cells represent dead cells. (**D**) Persistent bacteria exhibit a biphasic killing curve in response to the antibiotic killing and re-enriching the microbiota upon antibiotic removal. (Fig. 1A adapted from reference 49.)

Persister cells are a subgroup of bacterial populations that are highly tolerant to a variety of antibiotics, which can survive the treatment of infections by tolerating antibiotics, and can re-proliferate after the effect of antibiotics disappears, resulting in prolonged therapy and disease recurrence (Fig. 1D) [1]. The phenotypic characteristics of persisters have been clearly described: First, persisters had the same minimum inhibitory concentration (MIC) as the susceptible (Fig. 1B). Second, the percentage of persisters in the population is tiny (usually less than 1%). Third, persisters are a transient phenotypic transformation; unlike drug-resistant bacteria, the genetic material of persister bacteria does not change, and the persisters that survive antibiotic killing are recultured and show the same antibiotic susceptibility as their parents [49] (Fig. 1C). When faced with the threat of antibiotics, the susceptible bacteria in the microbiota are killed first, while the subpopulation of persisters dies relatively slowly, so the change curve of the number of living bacteria over time will first show a rapid decline, after all the susceptible bacteria die, the decline rate decreases abruptly and gradually converges to the level (biphasic killing curves) (Fig. 1A) [49]. Persistent bacteria are usually considered to be in a “dormant” state, their growth is slow or stagnant [11, 50], and their metabolic level is reduced [51], thus protecting cellular processes. When the deleterious effects disappear, the survival environment is once again suitable, and persisters can be used as a “spark” of the microbiota to enrich the microbiota. However, it has also been shown that the percentage of persistent bacteria is significantly higher in slow-growing dormant cells, but most of the dormant cells are not persisters [52], so it cannot simply be assumed that persisters are dormant cells.

### Antibiotic resistant cell and tolerant cell

Resistant cells are bacteria that carry resistance factors by altering their genetic material and gaining the ability to prevent antibiotics from binding to their intended targets, for example, by mutating antibiotic target genes, altering the permeability of cell membranes or using efflux pumps to reduce intracellular drug concentrations, and directly destroying antibiotic structures so that drug resistance can be passed on to the next generation (Fig. 1C) [53]. The MIC of resistant bacteria was significantly higher compared to susceptible bacteria (Fig. 1B), and the resistance factor allowed resistant bacteria to grow in the presence of antibiotics at MIC concentrations of susceptible bacteria. Resistant bacteria are in a dominant position in natural selection, and the constant generation of mutations gives some resistant bacteria new resistance, generating multidrug-resistant bacteria, and even superbugs that are resistant to most antibiotics, which is undoubtedly a significant threat to global public health security.

Tolerant cells are bacteria that survive exposure to bactericidal antibiotics for a period of time. Unlike resistant bacteria, tolerant bacteria do not carry resistance factors and do not have an elevated MIC (Fig. 1B), but die significantly slower than susceptible bacteria at lethal concentrations of antibiotics (Fig. 1A and C) [49]. In short, killing resistant bacteria requires a longer duration of antibiotic action rather than a higher concentration of antibiotics. Similar to persistence, bacterial tolerance is often caused by slow bacterial growth [54], and both can survive for prolonged periods at concentrations of bactericidal antibiotics above the MIC, but persisters are a smaller proportion of subpopulations in the microbiota, whereas tolerance is for the microbiota as a whole [55].

### Viable but non-culturable state

Viable but non-culturable (VBNC) cells are thought to be a survival strategy for bacteria in the face of adverse environmental changes, and bacteria can switch from an active to a VBNC state when stressed by environmental factors [56]. Bacteria that enter the VBNC state survive in a near dormant state and generally cannot grow on standard media, but have significantly improved tolerance to multiple antibacterial drugs [56, 57]. VBNC are similar to persisters in that both can survive environmental stress in a low metabolic state and are tolerant to a variety of antibiotics, but when the environment is restored to a condition suitable for bacterial growth, persisters will gradually recover and proliferate, while the recovery of VBNC often requires the stimulation of some specific factors, such as pyruvate and glutamate to promote the conversion of VBNC to a culturable state [58, 59]. Therefore, it has been suggested that the persisters and VBNC are homogeneous bacterial subgroups at different “dormancy depths“ [60].

## Molecular mechanism of persister formation

There is no conclusive evidence on the mechanism of persister formation, but in recent years studies have shown a strong link between persister formation and the toxin-antitoxin (TA) systems, SOS response, Stringent response, (p)ppGpp signaling, adenosine triphosphate (ATP) depletion, biofilm, and quorum sensing (QS) bacterial communication.

### Toxin-antitoxin systems

**Fig. 2:**
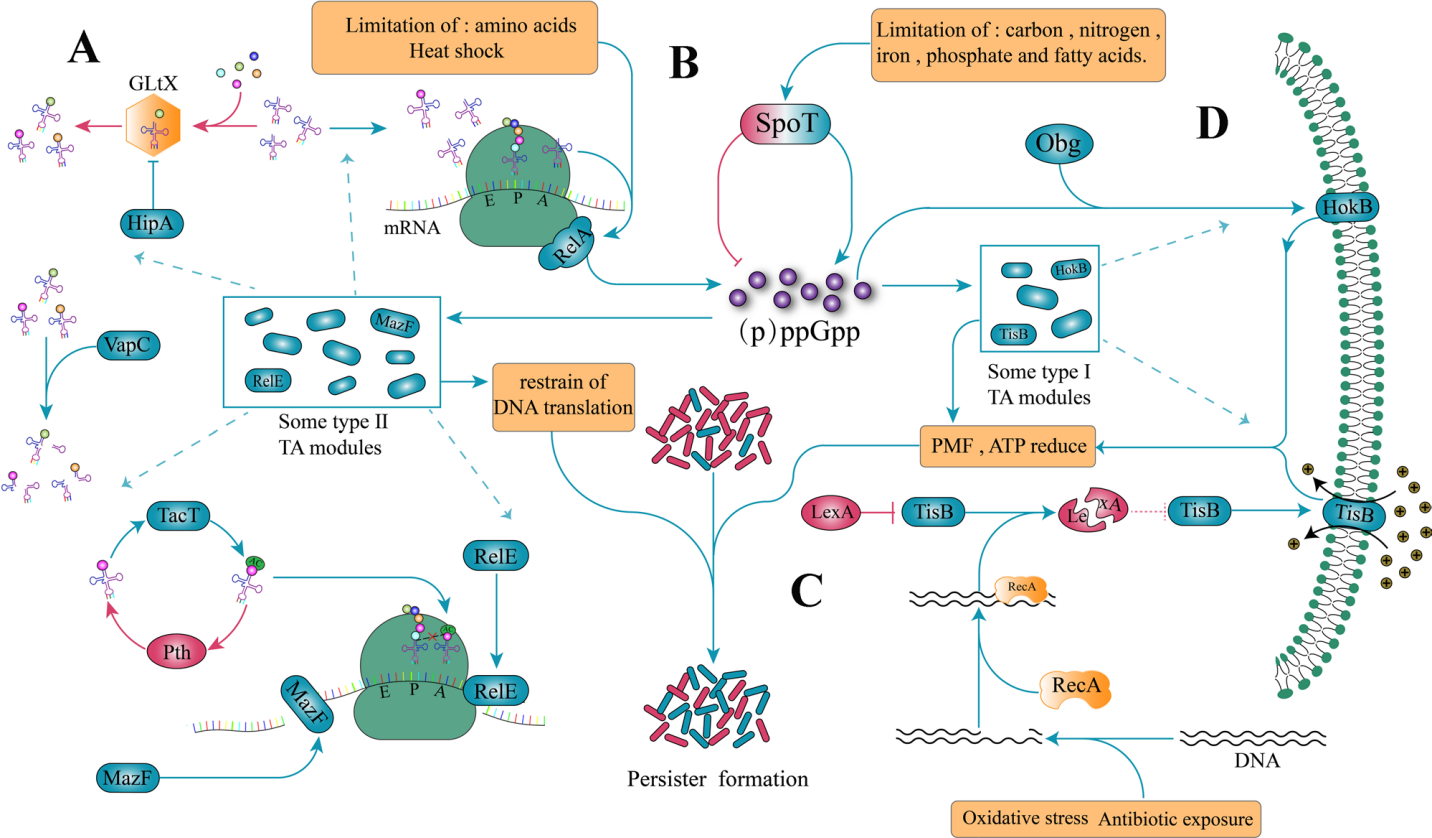
The main molecular mechanisms that induce the formation of persisters. (**A**) Most of the toxins in the type II TA modules can act as translation inhibitors, inhibiting cellular translation and inducing the formation of persisters by cutting mRNA, cutting tRNA, phosphorylating amino acid tRNA synthetase, modifying tRNA molecules, and many other ways. (**B**) The Stringent response can be triggered by various factors such as pH change, heat shock, and nutrient restriction, and is mainly mediated by (p)ppGpp. (p)ppGpp is produced by RelA or SPoT; it can affect the formation of persisters by modulating multiple type I and type II TA modules; however, in the absence of triggering factors, SPoT can inhibit (p)ppGpp production, keeping (p)ppGpp at a low level. (**C**) Cellular DNA damage induced by antibiotic exposure, oxidative stress, etc. The single-stranded DNA produced by the damaged DNA replication will activate the SOS response, which in turn will lead to the toxins of certain type I TA modules exerting their toxic effects. (**D**) The toxin of the type I TA modules can be inserted into the cell membrane and disrupt the proton-motive force that inhibits ATP synthesis. The rounded blue rectangles in the figure represent toxins (their corresponding antitoxins are not shown), the blue arrows represent facilitated persistence, and the red arrows represent diminished persistence

The toxin-antitoxin module consists mainly of stable toxins and unstable antitoxins cognate to them. Under normal conditions, toxins and antitoxins are kept in balance to maintain the normal physiological activities of cells [61]. The TA module is often described as a stress response element that is activated under stress [62], where partially unstable antitoxins are selectively degraded under the pressure of the survival environment, and the toxins exert their toxic effects interfering with critical cellular processes, leading to stagnation and dormancy of cells [63]. The TA system can be classified into six types according to the mechanism of neutralization of toxins by antitoxins [64]; it is now generally accepted that type I and type II TA modules, especially type II TA modules are closely associated with persister formation [62, 64, 65]. In *E. coli*, toxins in the type I TA modules *TisB/istR*, *hokB/sokB*, and *Ghot/GhoS* can insert into the bacterial inner membrane and thereby disrupting the proton-motive force that inhibits ATP synthesis and promotes persister formation (Fig. 2D) [66, 67]. The first gene identified to directly affect persistence is *hipA* from *E. coli*, which encodes the toxin portion of the type II TA module HipAB [68]. The *hipA* mutant *hipA7* could increase the HipA: HipB ratio by affecting HipA to promote persister formation [68, 69]. HipA is a serine-threonine kinase that phosphorylates the aminoacyl tRNA^glu^ synthetase GltX, the accumulation of uncharged tRNA at the ribosomal A site leads to the activation of RelA and an increase in (p)ppGpp alarmone (Fig. 2A) [70, 71], and (p)ppGpp regulates persister formation (see below). A related study showed that *hipA7* mutants were selected in patients with recurrent urinary tract infections, providing strong evidence for a correlation between the *hipA* gene and persisters [5]. Similar to the action of HipA, a variety of type II TA modules can promote the production of persistence by inhibiting cellular translation (Fig. 2A). VapC can inhibit cellular translation by cleaving rRNA or tRNA molecules [72]; TacT can modify the primary amine group of amino acids charged onto tRNA molecules and block the formation of peptide bonds, thereby inhibiting translation. The activity of TacT on translation is counteracted by the action of Pth, which promotes the recovery of persisters [73]. RelE exhibits specific cleavage of mRNA high codons through binding to ribosomes [74, 75], and MazF can inhibit translation directly by cleaving mRNA and indirectly by reducing translation factors and promoting ribosomal dormancy [76]. In *E. coli*, overexpression of translationally inhibitable toxins (RelE and MazF) increases the proportion of persisters [64, 77].

Although many studies have reported the relationship between the toxin-antitoxin system and persisters, its role in persister formation remains controversial. Either reduction of ATP synthesis or inhibition of translation slows cell growth, and slow-growing cells exhibit greater antibiotic tolerance. Therefore, any factor that contributes to slowing cell growth (e.g., overexpression of most toxins) would be expected to increase persistence. Vazquez et al. [78]. showed that overexpression of unrelated proteins, such as DnaJ and PmrC, also reduced cell growth and that cells overexpressing these proteins had higher survival rates when challenged with ciprofloxacin and ampicillin, which is similar to the results of overexpressing certain toxins, such as HipA [78]. Unfortunately, biphasic killing curves were not determined in this study, making it impossible to directly attribute increased survival to increased persistence [78]. It should be noted that Goormaghtigh and colleagues [79]knocked down 10 TA modules in *E. coli* and found no significant effect on their persistence, which could be explained by the very large functional redundancy of the TA modules on persistence. It is now well established that the toxin-antitoxin system acts as a stress response module that shares similar upstream triggers with persister formation: e.g., nutrient depletion and pH changes [80]. As to whether the toxin-antitoxin system plays a regulatory role in persister formation requires further study.

### Stringent response and (p)ppGpp signaling

The stringent response is a stress response triggered mainly by nutrient restriction (amino acid or fat depletion, etc.) and is regulated by both guanosine tetrakisphosphate (ppGpp) and guanosine pentakisphosphate (pppGpp). (p)ppGpp synthase RelA is activated by amino acid starvation and heat stress [81], while SpoT has a dual function of synthesis and hydrolysis, carbon, nitrogen, phosphate, iron, and fatty acid starvation will activate SpoT to produce (p)ppGpp [82], while under favorable conditions SpoT will keep (p)ppGpp at low levels (Fig. 2B) [81]. In *E. coli*, (p)ppGpp directly inhibits DNA primase and also targets a variety of proteins involved in lipid metabolism and nucleotide metabolism [83, 84], and can inhibit rRNA synthesis by regulating the transcription of the ribosomal regulatory factor (Rmf), which in turn affects global cellular translation [85]. (p)ppGpp also regulates the TA system; for example, increased levels of (p)ppGpp due to amino acid starvation will upregulate multiple type II TA modules, including *hipAB*,* dinJ/yafQ*,* mazEF*,* mqsRA*,* relBE*, and *yafNO* [27, 86, 87], as well as the type I TA module *hokB* (Fig. 2B) [66]. Mutants lacking *relA*, *relA* and *spoT* typically form fewer persisters due to reduced levels of (p)ppGpp [88]. In conclusion, (p)ppGpp can reprogram cellular life activities to adapt to harsh survival environments through transcriptional reprogramming and direct regulation of target protein activity, and these changes will result in slow cell growth or dormancy.

### SOS response

When bacteria are exposed to various adverse environments (oxidative stress, extreme pH, bactericidal antibiotics), they are prone to DNA damage, which in turn triggers the SOS response [50, 89]. When DNA damage occurs, RecA proteins are activated by the single-stranded DNA produced by processing the damaged DNA, inducing the SOS response (Fig. 2C) [90]. The role of the SOS response in persister formation has been reported in several species, including *Escherichia coli* [91], *P. aeruginosa* [92], *Staphylococcus aureus* (*S. aureus*) [93], and *Mycobacterium tuberculosis* (*M. tuberculosis*) [94]. The SOS response serves as a stress signaling pathway that activates type I (*tisB/istR-1*, *symE/symR*, *diQ/agrB*, *hokE/sokE*) and type II (*yafNO*, *dinJ/yafQ*) TA modules, allowing toxins to exert their toxic effects and inducing the production of persisters [90, 91, 95–97]. For example, in *E. coli*, the SOS response can cause the cleavage of the SOS gene blocker LexA, which inhibits the toxin TisB of the *tisB/istR-1* module, thus causing the accumulation of free TisB. TisB is a small hydrophobic peptide, and the insertion of TisB into the inner membrane depolarizes the inner membrane, disrupts the proton-motive force that inhibits ATP synthesis, and induces bacterial dormancy and persister formation (Fig. 2C) [98, 99]. The effect of the SOS response on persistence was also demonstrated by the reduced production of persisters in the *tisB* deficient strain and the increased proportion of persisters in the *istR-1* deficient strain under the influence of antibiotic stress [91, 100].

### Impact of global regulators on persisters

Several recent studies have shown that global regulators can affect the production of persisters in different ways. Obg is a GTPase that regulates ribosome function and is involved in DNA and protein synthesis. Verstraeten and his team showed that a central role for Obg in determining persistence in response to nutrient starvation [66]. In *E. coli*, Obg, with the involvement of (p)ppGpp, affects the transcription of the type I toxin-antitoxin module *hokB-sokB* [66, 101], and increased levels of Obg induces the expression of HokB, which has similar properties to TisB, both of which insert into the cell membrane leading to PMF disruption and decreased ATP synthesis, inducing the formation of persisters [66]. PhoU is a negative global regulator that, beyond its role in phosphate metabolism, facilitates persister formation by suppressing many critical cellular metabolic processes [102]; PhoU deficiency results in increased cellular metabolic activity, increased expression of energy-producing genes, and reduced persistence [102]. RpoS is a global regulator that plays a vital role in the general stress response that is triggered by nutrient deprivation, temperature changes, pH extremes, oxidative stress, etc. It is a key molecular mechanism that enables bacteria to survive under pressure; RpoS can replace σ-factors in the transcriptional reprogramming of bacteria and generally increase the stress tolerance of the population [103]. Upregulated RpoS expression has been found under stress conditions, accompanied by increased population persistence [104]. Recent studies by Kim Lewis’ team have shown that the integration host factor (IHF) reduces cellular ATP levels by regulating the expression of key enzymes at the junction of the tricarboxylic acid (TCA) cycle and the glyoxalate bypass, shifting carbon from efficient energy production to carbon conservation pathways and thereby promoting the formation of persisters [105]. In *Salmonella*, Fis regulates persistence by modulating glutamate metabolism [106]. Similarly, in *Salmonella typhimurium*, the RNA-binding protein ProQ was reported to be a global regulator of gene expression, which promotes the formation of persistent bacteria by activating energy-consuming processes that include flagellar motility and protein secretion [107]. The global regulators reported so far can affect persistence in many ways rather than through a defined pathway, suggesting that complex, diverse, and redundant regulatory mechanisms affect persistence.

### ATP depletion

The TA systems, as well as the general stress response, were thought to be the best possible candidate to explain the persistence phenomenon; however, this speculation has recently been challenged [41, 79]. Based on the phenomenon of low nutrient levels during the plateau phase of bacterial growth and a significantly higher proportion of persisters during the plateau phase than during the exponential phase, the researchers propose the theory that fluctuations in ATP depletion mediate the production of persisters. In *S. aureus*, Eliza A. Zalis and colleagues constructed several key TAC cycle enzyme deficient strains and demonstrated that the TAC cycle enzyme deficient strains were significantly more capable of forming persisters than wild-type strains and that the high persistence strains had lower ATP levels [108]; in contrast, knocking out the TA module and (p)ppGpp synthase in *S. aureus* had no significant effect on persistence [41]. Natural fluctuations in the expression levels of genes controlling energy production have been reported to influence the formation of *M. tuberculosis* persisters [109]. Similar results were found in *E. coli*, where a reduction in ATP levels by arsenate treatment increased persistence [27]. Similarly, treatment with cyanide-m-chlorophenylhydrazone (CCCP) (a metabolic inhibitor) significantly increased the proportion of persisters in the colony [110]. ATP levels have not been found to affect persistence through a specific pathway, and it is speculated that lower intracellular ATP levels may down-regulate critical central cellular physiological processes (e.g., transcription, translation), leading to inactivation of antibiotic targets, and thus multi-drug tolerance in bacteria. Besides, a recent study has shown that reduced ATP levels lead to the accumulation of insoluble proteins in the cell, promoting the transformation of sensitive cells into persisters, which may also be one of the reasons why ATP affects persistence levels [60].

### Bacterial communication

Quorum sensing (QS) is a bacterial communication network that allows bacteria to transmit information by secreting autoinducers as signaling molecules that manage the investment of community resources into phenotypically distinct subpopulations, allowing colonies to exhibit uniform behavior conducive to population survival in adapting to their environment, resisting hazards, and competing with other species [111–114]. There is increasing evidence that QS, as well as intercellular chemical signaling molecules, play an essential role in establishing heterogeneity in bacterial populations [115–119]. Suppose persisters are considered to be strategies used by bacterial populations to enhance the colony’s response to environmental risks and to ensure that the colony does not go extinct in the event of a sudden environmental change; in that case, then it is clear that QS, as the language of bacteria, has a regulatory role in the formation of persisters. For example, in *S. aureus*, the Agr quorum sensing system represses persister formation through regulation of phenol soluble modulins [115]; *P. aeruginosa* responds to the population-sensing signal molecule acyl-homoserine lactone to increase the proportion of persisters in the colony [120]; the stress-inducible quorum-sensing peptide CSP (competence-stimulating peptide) can induce the formation of *Streptococcus pyogenes* persisters [118, 121].

Similarly, intercellular signaling molecules have been shown to be involved in the regulation of persistence. Indole is an intercellular signaling molecule that, unlike QS signaling molecules, can regulate bacterial population behavior through either persistent or pulsed signaling modes [122]. Whereas QS regulates gene expression in response to fluctuations in cell population density, QS signaling molecules increase with cell density, and cells sense their concentration to regulate gene expression [123]. In particular, the role of indoles in the formation of persisters is the subject of much controversy. The results of Nicole M. Vega et al. showed that in *E. coli*, the proportion of persisters incubated with indole (500 µM) increases by at least an order of magnitude [124], *tnaA* encodes a protein that converts tryptophan to indole. *tnaA* deficient strain contains very little indole and is almost an order of magnitude less able to form persisters [124]. The results of Manon Lang et al. also showed that indole (350µM) increased the persistence of *Vibrio cholerae* to aminoglycoside antibiotics [24]. On the contrary, some other findings suggest that indole (1–2 mM) may reduce persistence [125, 126], and 1–2 mM is considered a toxic concentration [24]. In summary, we believe that the effect of indole on persistence may depend on its concentration.

### Bacterial biofilms

**Fig. 3:**
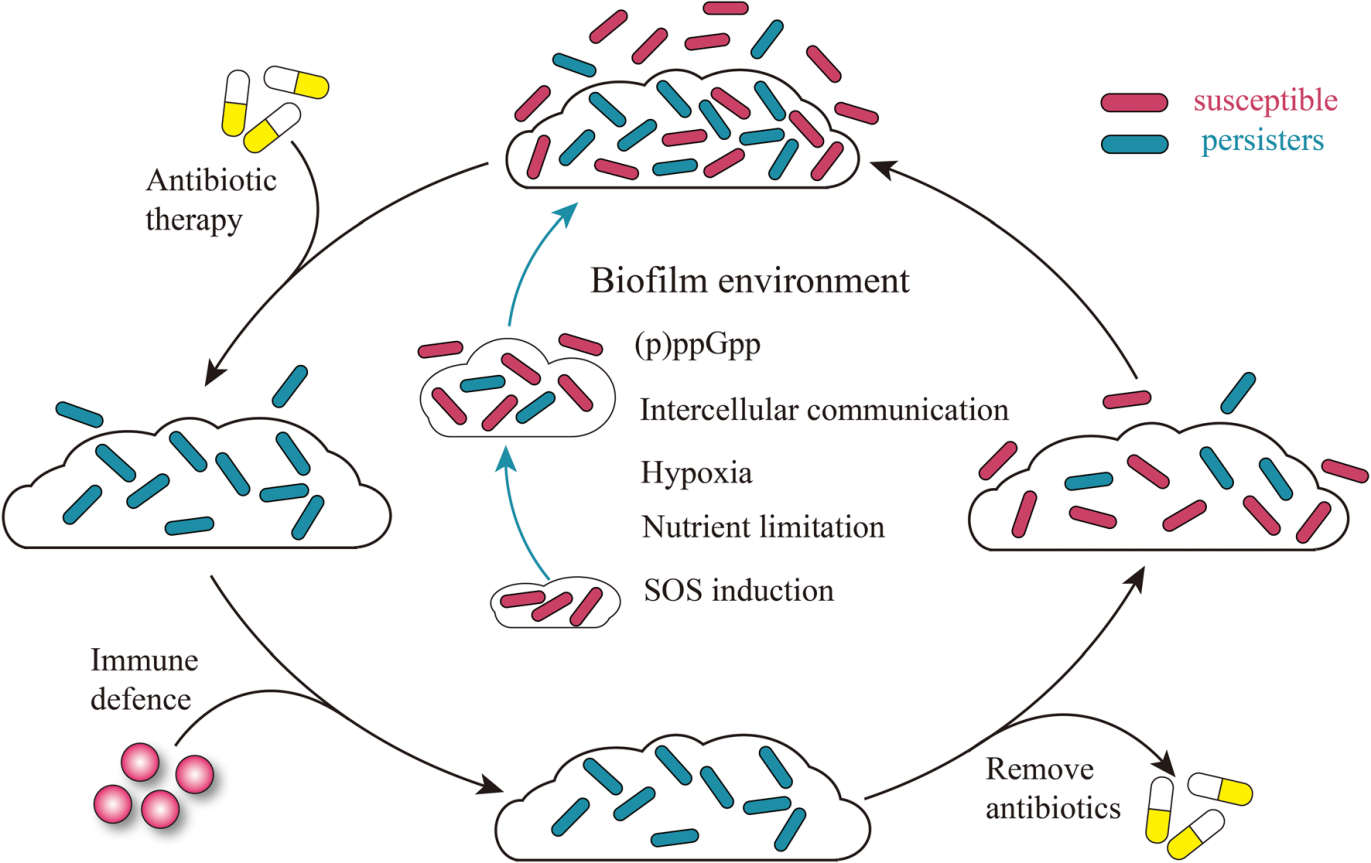
A model of biofilms promoting the production of persisters and causing persistent infection. When the biofilms begin to form, bacteria within the biofilms are subjected to environmental conditions such as nutrient limitation and hypoxia, triggering the stringent response and the SOS Response, leading to an increase in persistent bacteria within the biofilms, and intercellular communication further facilitates this process. In the face of antibiotic treatment, susceptible bacteria inside and outside the biofilms are killed, and the organism’s immune defense can kill the persisters outside the biofilms, but the action of the biofilm hinders the killing of the persisters inside the biofilms by the immune defense, and when the antibiotic treatment disappears, the surviving persisters inside the biofilms will recover and re-enrich the bacterial population

Bacterial biofilms refer to the proliferation and differentiation of bacteria adhering to inert or active surfaces formed by communities embedded in a self-produced matrix of extracellular polymeric substances [127]. Biofilms can block a variety of bactericidal antibiotics and other immune substances, protect the bacteria from a variety of adverse conditions, including immune response, and improve the survival of the bacteria [128, 129]. However, some antibiotics can easily penetrate the biofilms and still can not achieve an excellent bactericidal effect [130]; therefore, the higher tolerance of biofilms to antibiotics has been attributed to the persisters within them [130–133]. The biofilm matrix surrounds the cells, providing a protective barrier while impeding the cell’s access to nutrients and oxygen. Hypoxia and nutrient depletion can promote the formation of persisters through several different mechanisms e.g. (p)ppGpp, intercellular communication, and SOS induction [134]. Different research groups have detected the enrichment of persisters under a variety of common bacterial biofilms [4, 135–137]. Lewis proposed the model that biofilm causes the recurrence of infection [1, 138], and based on this; we added that the biofilm environment promotes the formation of persisters through different mechanisms. Figure 3 depicts the model that biofilm promotes the enrichment of persisters and causes the recurrence of infection (Fig. 3) [1, 138].

## Approaches and methods for studying persistence

### Detection of antibiotic persistence

According to the present study, no specific molecular markers or phenotypic characteristics of the persisters were found to distinguish the susceptible bacteria, and the distinction of persistence was mainly based on the time-kill assay, which determines the survival of bacteria at different time points during antibiotic exposure [55]. Survival is defined as the ability to regrow after antibiotic removal. The hallmark of persistence is the bimodal (or multimodal) killing curve, which represents the presence of two (or more) subgroups of cells with different susceptibilities to antibiotics (Fig. 1A). However, to confirm that one of the subgroups is persisters, effects such as drug-resistant mutants, tolerance phenotypes, and changes in the killing capacity of the drug with time degradation should also be avoided [49].

### Methods for the enrichment of persisters at the population level

In the natural state of actively growing microbiota, the proportion of persisters is small, they are not heritable and lack a unique molecular identity, making it difficult to accurately separate persisters from susceptible and drug-resistant bacteria and to obtain sufficient samples for research [28, 55]. Therefore, specific methods can be used to induce the generation of persisters to increase their proportion in the microbiota.

**Fig. 4:**
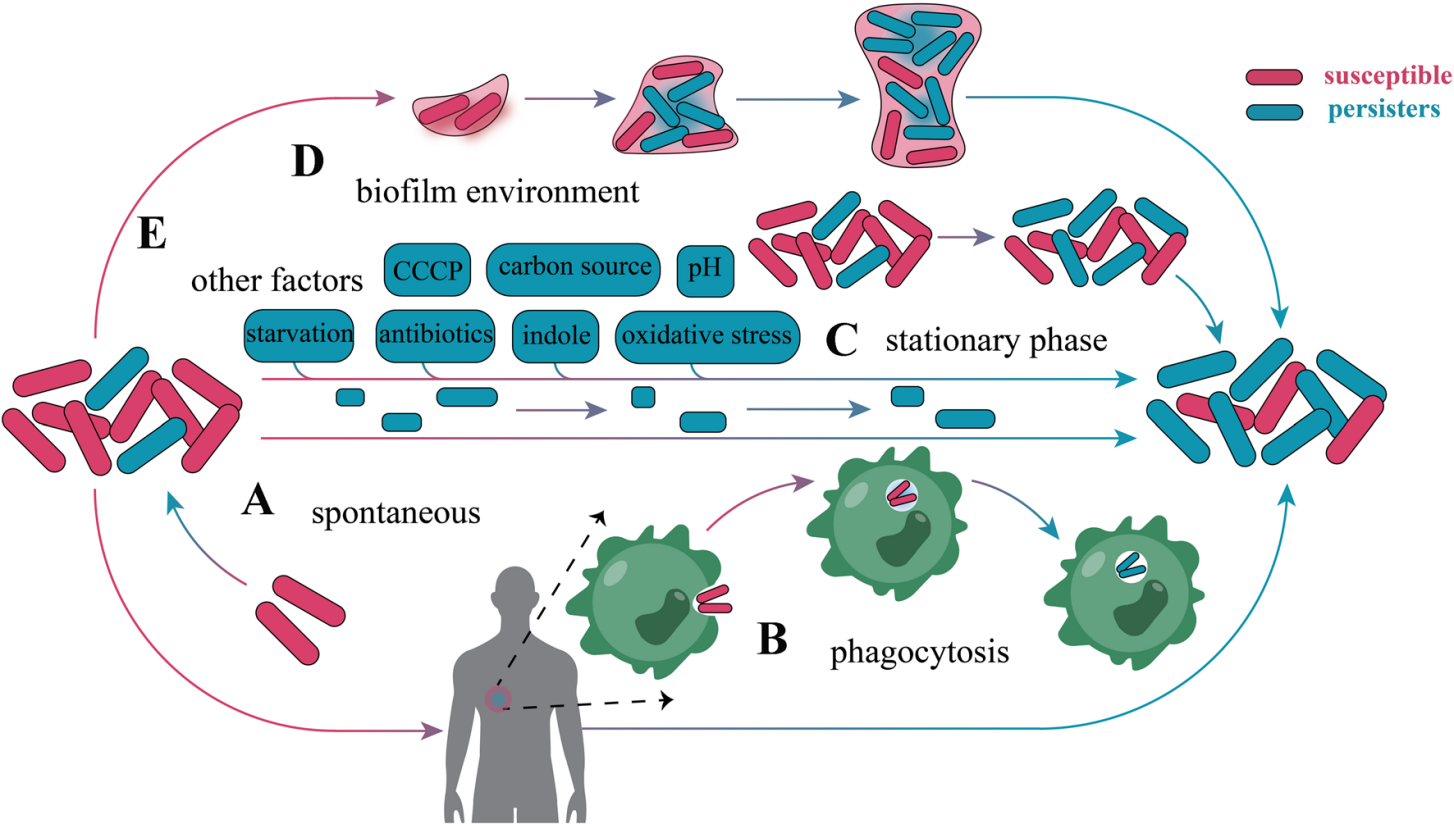
Different factors that promote persister formation. (**A**) Part of the exponentially growing microbiota is randomly converted into persisters. (**B**) In the host, phagocytosis of immune cells promotes the formation of persisters. (**C**) The proportion of persisters in the stationary phase microbiota increases significantly. (**D**) Biofilm environment encourages the conversion of normal microbiota into persisters. (**E**) Antibiotic treatment, starvation, pH changes, oxidative stress, carbon source conversion, and treatment with metabolic inhibitors have promoted the transformation of normal microbiota to persisters

As a bacterial survival strategy in response to environmental stress, persisters can form randomly in normally growing colonies or be induced by various factors (Fig. 4). Depending on the mode of formation, persisters can be classified as Type I or Type II persisters, also known as triggered persisters or spontaneous persisters [49]. When the bacteria are in a steady state of exponential growth, a part of the population will be converted into persisters at a constant rate, called Type II persisters. The production of Type I persisters requires specific triggers, which are usually environmental stresses that are detrimental to the microbiota, such as biofilm (Fig. 4D) [139], phagocytosis of immune cells (Fig. 4B) [140], nutrient starvation [141], pH changes [142], oxidative stress [143], antibiotic treatment [1] (Fig. 4E). The level of Type I persisters is related to the nature and duration of the environmental stress.

Nutrient starvation is one of the most common environmental factors that promote persister formation, where bacterial nutrient levels are lower during the plateau growth phase and the proportion of persisters in the colony is significantly higher than during the exponential growth phase [134]. Therefore, reducing nutrient levels by prolonged incubation is an easy way to enrich persisters. In a long-term culture test of *E. coli*, it was found that the proportion of persisters was significantly higher in 24-hour cultures compared to exponential growth period cultures [60]. In addition, some metabolic inhibitors can significantly increase the proportion of persisters in the colony; for example, CCCP or arsenate incubation significantly increased the proportion of persisters in *E. coli* [60], *S. aureus* [41]and *P. aeruginosa* [48].

**Fig. 5:**
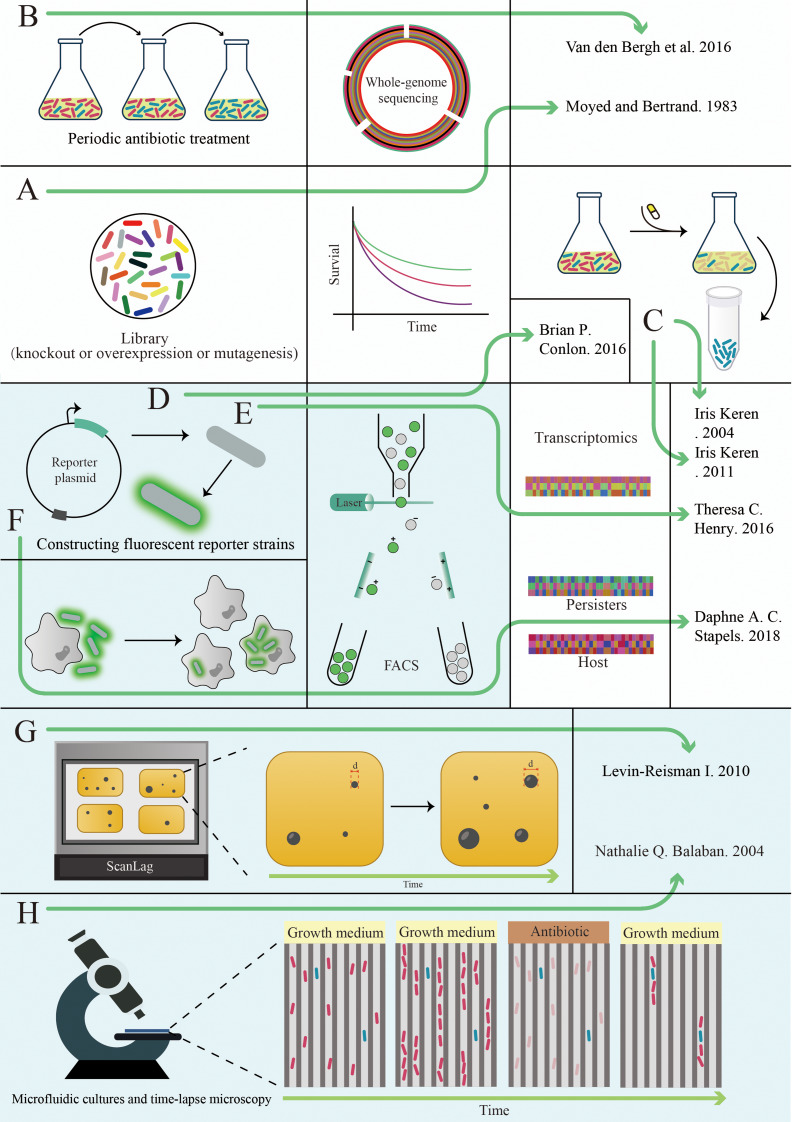
Main methods used to study persisters. We list a selection of articles that either applied these methods to persister studies for the first time or made important advances in persister studies using these methods, and indicate the combinations of methods they used with a green line, with single-cell techniques shown in light green background panels and non-single cell techniques shown on a white background. (**A**) Using an antimicrobial drug-induced approach, the Lewis team [68] screened for mutant strains with significantly increased persistence and localized the mutant genes. (**B**) Jan Michiels’ team [146] discovered the rapid evolution of persistence in E. coli during repeated antibiotic treatments and used whole-genome sequencing to show that this evolution was driven by a single-point mutation in a few genes. (**C**) Similarly, the Lewis team [144, 145] uses antimicrobial agents to lyse sensitive bacteria and collect persisters by centrifugation for subsequent studies. (**D**) The Lewis team [41, 108] combined fluorescent reporter protein genes with energy production-related genes and found that persister formation was associated with a decrease in ATP levels by FACS analysis. **E**) Based on the construction of fluorescence-expressing strains, Theresa C. Henry’s team [147] used FACS in conjunction with transcriptomics to study the physiology of persisters. (**F**) Sophie Helaine’s team [140] used fluorescent protein expressing strains to infect host cells, which were sorted by FACS, and then used dual RNA-seq to simultaneously detect transcriptome changes in both the persisters and host cells, and found that the persisters had a disruptive effect on host immunity during infection. (**G**) Balaban’s group [148] has developed a method of monitoring changes in colony appearance that can be used to study the time it takes for persisters to return to their normal proliferative state. (**H**) Balaban’s team [26] uses microfluidic culture for the first time to study persisters.

Enrichment of persisters can be achieved by using antibiotics to lyse growing bacteria. For example, *E. coli* is treated with ampicillin, and the surviving persisters are collected by centrifugation [144]; and the lysis of *M. tuberculosis* by D-cycloserine enables the enrichment of persisters (Fig. 5C) [145]. Enrichment of persisters by antibiotic lysis of growing bacteria is simple and reproducible, and the harvested persisters can be used for downstream analyses such as transcriptomics. However, not all antibiotics lyse bacteria after killing them. In addition, exogenous antibiotics affect the expression of bacterial genes, and it cannot be excluded that the detected differential genes are the result of antibiotic action. However, due to the lack of methods to enrich for persisters without interfering with bacterial growth, the similar transcriptome results are still of high research value.

### Application of single-cell technology in the investigating of persisters

#### Flow cytometry

Flow cytometry is a technique for the rapid characterization of small biological particles, such as bacteria. It is an advanced method commonly used in biological research to characterize these biological features at the single-cell level by rapidly detecting photons emanating from individual cells and thus quantifying various essential parameters such as size, granularity (the material inside the cell), and various fluorescent properties of the cell.

The advent of fluorescence-activated sorting has provided a major boost to the development of flow cytometry. Cells with a desired fluorescence characteristic can be differentially charged and isolated using electrical currents and electromagnetic devices. This technique plays an important role in the study of bacterial phenotypic heterogeneity by allowing the isolation of single cells for subsequent studies. In the absence of techniques to separate persistent bacteria from other cell types, fluorescence-activated cell sorting (FACS) has become the gold standard for examining persisters’ physiology [52, 149]. Mohiuddin and colleagues use fluorescent cell-activated sorting combined with a fluorescent protein expression system to monitor the recovery process of persisters at the single-cell level [150]. Eliza A. Zalis et al. linked the green fluorescent protein gene to several TCA cycle enzyme genes and used fluorescence-activated cell sorting to achieve enrichment of persistent bacteria, confirming that persister formation is associated with ATP levels regulated by the TCA cycle (Fig. 5D) [108]. Vivek Srinivas and colleagues developed the PerSort system based on fluorescence-activated cell sorting to isolate *M. tuberculosis* persisters from normally growing *M. tuberculosis* by measuring cell translation levels [151], Similarly, Theresa C. Henry et al. developed Persister-FACSeq by combining FACS and next-generation sequencing technologies and applied it to study persister physiology and its heterogeneity(Fig. 5E) [147]. To obtain more information about cell morphology, flow cytometry can be combined with fluorescence microscopy, a technique known as imaging flow cytometry (IFC), which provides fluorescence-indicated morphological and physiological information in the context of a single cell by capturing multiple images as the cells pass through, compared to traditional flow cytometry. The high-throughput information collection and image capture capabilities of IFC have been effective in studying the physiological properties of quiescent cells [152] and in monitoring the recovery of persisters after antibiotic treatment [18]. Much of the difficulty in studying persisters lies in the inability to obtain sufficient samples for study, as persisters are subpopulations of the flora and represent a relatively small proportion of the population. Fluorescence-activated sorting, which allows the physical isolation of persisters, has advanced the development of persister research. However, it cannot be ignored that this method has some limitations. First, fluorescence-activated sorting is highly dependent on specific fluorescent markers, but relatively few fluorescent reporter genes have been successfully used to characterize and isolate persisters [108, 151, 153–155]. In addition, the process of fluorescence-activated sorting is complicated and tedious, while the physiological characteristics of persisters are reversible, and the characteristics of persisters may change during the sorting process.

#### Microfluidic cultures and time-lapse microscopy

Microfluidic devices have greatly improved the ability of microscopy to analyze persisters, allowing individual cells to be monitored for long periods under ideal bacterial growth conditions [124]. Microfluidic devices allow monitoring of the process by which persisters revert to sensitized bacteria and begin to proliferate, providing a new perspective on controlling persistent infections caused by persisters. Persistent infections can be controlled by preventing persisters from recovering and proliferating, or by inducing them into deeper “dormancy depths“ [60]. Balaban et al. were the first to apply microfluidic culture to the study of persistence and observed type I and type II persisters of *E. coli* at the single-cell level for the first time(Fig. 5H) [26], then Orit Gefen and colleagues used a microfluidic device to monitor the synthesis of fluorescent proteins under a synthetic promoter to characterize the dormancy of individual persisters and found that exposure of bacteria to antibiotics at specific stages significantly reduced persistence [156]. Nicole M Vega et al. used microfluidic culture to monitor indole-induced persistence formation and determined the role of oxidative stress and phage shock pathways in this phenomenon [124]. Subsequently, Yuichi Wakamoto et al. combined microfluidic culture with time-lapse microscopy to monitor the killing of *Mycobacterium smegmatis* by isoniazid (INH) at the single-cell level and found that *Mycobacterium smegmatis* produces persisters by division in the presence of INH [11]. Based on time-lapse imaging, Balaban’s team developed the ScanLag technology, which measures in parallel the delay in growth (lag time) and growth rate of thousands of cells; this has important implications for monitoring persisters’ formation and recovery process(Fig. 5G) [148]. Similarly, with the rapid development of microscopic resolution, Yingying et al. used high-throughput light microscopy to monitor the recovery process of persisters. They found the existence of “dark foci” (protein aggregates) in persisters and the difference in the time required for recovery of persisters which led to the concept of “dormancy depth.” and demonstrated that dynamic protein aggregation regulated by ATP can regulate the dormancy depth of bacteria and significantly affect the tolerance of bacteria [60]. Microfluidic devices and time-lapse microscopy allow monitoring of individual cells over long periods of time, but the higher accuracy limits the number of targets that can be monitored simultaneously. However, time-lapse microscopy makes it difficult to accurately find persisters due to the small percentage of persisters in the colony. This problem can be solved by combining flow cytometry for initial isolation of persisters, followed by monitoring and analysis of individual cells using microfluidic culture and time-lapse microscopy.

#### Single-cell Raman Spectroscopy

Single-cell Raman Spectroscopy (SCRS) is a non-invasive technique for characterizing the physiological properties of individual cells, which uses a monochromatic laser as a light source to penetrate cells to obtain cellular Raman spectra [157]. When laser light is transmitted to proteins, lipids, sugars, and other substances in the cell, the scattered light signal can be expressed as different specific spectra, which are like molecular “fingerprints” of particular substances, and the full spectrum of the cell can reflect the physiological state and intrinsic characteristics of the cell. SCRS can be used for many studies, such as the detection of cellular metabolites [158], the detection of cellular metabolic state, the determination of bacterial antibiotic resistance [159, 160], and the characterization of single-cell phenotypic diversity of microbial populations with non-destructive and label-free characteristics [161]. Currently, several reports describe the survival mechanism of persistence as entering a metabolically inactive state of growth inhibition under environmental stress, but recent studies by two different groups have reached different conclusions; Chuan Wang et al. used SCRS in combination with D_2_O labeling (D_2_O-Ramanometry) and found that *E. coli* persisters exhibited more vigorous metabolic activity [162]. Hiroshi Ueno used SCRS to analyze the metabolic activity of persisters under antibiotic treatment with rifampicin and confirmed that dormancy was not the cause of persister production in *M. tuberculosis* [12]. As persistence studies deepen, I believe more researchers will realize the advantages of SCRS, a non-invasive method that does not easily disrupt the phenotypic state of cells, making it a promising development in persistence studies.

### Advances in persistence genes

The search for genes associated with persister formation is one of the essential approaches to study bacterial persistence mechanisms. However, due to the non-heritable nature of persisters, the study of persistence-related genes was not initially emphasized until 1983 when Moyed and Bertrand used chemical induction and antimicrobial killing to screen for the first mutant strain with significantly improved persistence and localized the mutant gene in *hipA* [68]. Screening for strains with significant changes in persistence levels by constructing deletion, overexpression, or mutagenesis libraries became the first approach to studying persistence genes (Fig. 5A). Transcriptomics allows the analysis of global transcriptional changes in persisters. For example, using the effect of lysis of non-persistent *M. tuberculosis* bacteria by D-cycloserine and collection of surviving cells by centrifugation, and the transcriptomes of persistent bacteria can be obtained by hybridization to Affymetrix array [145]. RNA sequencing (RNA-seq) significantly increases the range and sensitivity of transcript detection compared to microarrays, which has greatly facilitated the development of transcriptomics [163]. Using dual RNA-seq methods, Helaine’s group sequenced the transcriptomes of *Salmonella* persisters and the host cells in which they reside during persistent infections(Fig. 5F) [140]. Michiels’ team reveals rapid evolution and adaptation of persistence during antibiotic treatment and uses whole-genome sequencing to locate mutant genes that cause high persistence(Fig. 5B) [146]. With the advancement of gene sequencing technology and the reduction of cost, next-generation sequencing (NGS) technology can provide a comprehensive analysis of genetic and transcriptomic information [164], especially single-cell sequencing technology can study rare genetic differences that are easily overlooked in populations and is suitable for studying phenotypic heterogeneity.

## Conclusion and prospect

In the past 20 years, with the rapid development of bacterial drug resistance, the study of persistent bacteria has gradually gained attention. In particular, during the treatment of cancer, a small proportion of cancer cells survive after drug treatment without resistance mutations, which exhibit reduced cell cycle kinetics and transient tolerance to toxic treatment [165, 166]. This way in which cancer cells adapt to environmental stress by changing their phenotype shows a high degree of similarity to persisters [167] and may therefore explain the emergence of both by similar mechanisms. The critical role of persistence in the recurrence of chronic infections, the generation of global AMR, and in cancer treatment has attracted great interest from researchers, and many advances have been made.

Current research suggests that the causes of persistence are complex and highly redundant and that no single mechanism can completely control the formation of persistent bacteria. Recent research has shown that multiple mechanisms, including toxin-antitoxin systems, SOS response, stringent response, and numerous global regulators, are involved in the formation of persistent bacteria by reducing cellular ATP levels, impeding DNA replication, inhibiting translation, and inducing protein accumulation in various forms. However, there are still many unanswered questions regarding the molecular mechanisms of bacterial persistence and its clinical manifestations, such as the upstream mechanisms for sensing environmental changes and making decisions are not clear; is the emergence of persistent bacteria in a suitable environment simply the result of random differences in gene expression levels? In the context of clinical treatment, how can the emergence of persistence in patients be measured in vivo? How do the host’s in vivo environment and immune system interact with persistent bacteria? How homogeneous are persistent bacteria in vivo versus in vitro? What is clear, however, is that phenotypic heterogeneity, influenced by multiple mechanisms, is critical for the survival of the microbiota.

Currently, it is widely recognized that single-cell technology plays an important role in the study of persisters. This is because further studies on persisters require their precise isolation. Emerging single-cell technologies such as fluorescence-activated cell sorting (FACS), imaging flow cytometry (IFC), microfluidic culture, and single-cell Raman spectroscopy (SCRS) are increasingly used in the study of bacterial phenotypic heterogeneity. However, it is undeniable that they still have limitations such as the lack of specific markers and the growth and complexity of the sorting process. It is believed that shortly, breakthroughs in advanced technologies will soon solve many of these problems that still exist in the study of persistence.

## Data Availability

No datasets were generated or analysed during the current study.

## Abbreviations

AMR: antimicrobial resistance
MIC: minimum inhibitory concentration
VBNC: viable but non-culturable cells
TA: toxin-antitoxin
QS: quorum sensing
ATP: adenosine triphosphate
TCA: tricarboxylic acid
CCCP: cyanide m-chlorophenylhydrazone
CSP: competence-stimulating peptide
FACS: fluorescence-activated cell sorting
IFC: imaging flow cytometry
INH: isoniazid
SCRS: Single-cell Raman Spectroscopy
NGS: next-generation sequencing
